# The interaction between cuticle free fatty acids (FFAs) of the cockroaches *Blattella germanica* and *Blatta orientalis*  and hydrolases produced by the entomopathogenic fungus *Conidiobolus coronatus*

**DOI:** 10.1371/journal.pone.0235785

**Published:** 2020-07-09

**Authors:** Agata Kaczmarek, Mieczysława Irena Boguś, Emilia Włóka, Anna Katarzyna Wrońska, Anna Krawiel, Michalina Kazek, Katarzyna Zalewska, Katarzyna Kłocińska-Biały, Martyna Sobocińska, Aleksandra Gliniewicz, Ewa Mikulak, Marta Matławska

**Affiliations:** 1 Witold Stefański Institute of Parasitology, Polish Academy of Sciences, Warsaw, Poland; 2 BIOMIBO, Warsaw, Poland; 3 National Institute of Public Health–National Institute of Hygiene, Warsaw, Poland; Al-Azhar University, EGYPT

## Abstract

The interactions between entomopathogenic fungi and insects serve a classic example of a co-evolutionary arms race between pathogens and their target host. The cuticle, site of the first contact between insects and entomopathogenic fungus, is an important defensive barrier against pathogens. It is covered by a layer of lipids that appears to play a key role in these processes and cuticular free fatty acid (FFA) profiles are consider as a determinant of susceptibility, or resistance, to fungal infections. These profiles are species-specific. The cockroaches *Blattella germanica* (Blattodea: Blattidae) and *Blatta orientalis* (Blattodea: Ectobiidae) are unsusceptible to the soil fungus *Conidiobolus coronatus* (Entomophthorales: Ancylistaceae) infection, therefore we studied the profiles of FFAs in order to understand the defensive capabilities of the cockroaches. The fungus was cultivated for three weeks in minimal medium. Cell-free filtrate was obtained, assayed for elastase, *N*-acetylglucosaminidase, chitobiosidase and lipase activity, and then used for *in vitro* hydrolysis of the cuticle from wings and thoraces of adults and oothecae. The amounts of amino acids, *N*-glucosamine and FFAs released from the hydrolysed cuticle samples were measured after eight hours of incubation. The FFA profiles of the cuticle of adults, and the wings, thoraces and oothecae of both species were established using GC-MS and the results were correlated with the effectiveness of fungal proteases, chitinases and lipases in the hydrolyzation of cuticle samples. Positive correlations would suggest the existence of compounds used by the fungus as nutrients, whereas negative correlations may indicate that these compounds could be engaged in insect defence.

## Introduction

Insect populations are regulated in part by the activity of entomopathogens. Entomopathogenic fungi are proposed as an eco-friendly alternative to chemical insecticides and as model organisms to study insect infection [1–4]. Unlike bacteria or viruses, fungi infect insects by direct penetration of the cuticle, followed by multiplication in the hemocoel [5].

Infection by entomopathogenic fungi is a multi-stage process comprising adhesion of fungal spores to the insect cuticle, germination and the penetration of invasive hyphae into the host body, hyphae propagation inside the hemocoel and colonization of the host internal organs, followed by the release of toxic secondary metabolites, which might result in host death [6]. The fungus penetrates the insect cuticle by a combination of mechanical pressure from growing hyphae and the enzymatic degradation of the proteins, chitin and lipids comprising the cuticle: proteases are produced first, followed by chitinases and lipases [7,8].

Two key factors influencing the infection process are the structure and composition of the host exoskeleton, and the efficiency of the immune system. Since the cuticle is the first point of contact between the insect and fungus, it is the first and most decisive defence mechanism in insects, and its composition varies greatly according to the species and the developmental stage [9–13]. This complex structure is covered by a waxy layer rich in lipids which play a key role in resistance to entomopathogenic fungi [8,14]. However, although many cuticular lipids have antimicrobial properties, other stimulate the germination process, growth and virulence of fungi; and variations in lipid profiles between species are reflected in differential susceptibility to infection [15–21]

The fungal proteases, chitinases and lipases used to degrade cuticle components play crucial roles in the infection process and are known to act in a coordinated fashion [5,22–24]. Some cuticular proteins display protease inhibition, and protect the insect by suppressing conidial germination and penetration [25,26]. Although no lipase and chitinase inhibitors have been identified in the cuticle so far, several natural chitinase and lipase inhibitors, mostly of microbial origin, have been described [9,27]. Further studies might bring more information on the presence of substances tempering the activity of fungal chitinases and lipases in the insect cuticle.

Previous studies on four medically-important fly species (*Lucilia sericata*, *Calliphora vicina*, *Calliphora vomitoria* and *Musca domestica*) identified correlations between the efficiency of cuticle digestion by fungal enzymes and the content of cuticular free fatty acids (FFAs), free fatty acid methyl esters (FAME), fatty alcohols, n-alkanes, sterols and several non-typical compounds [28].

The German cockroach (*B*. *germanica*), and the oriental cockroach (*B*. *orientalis*) are two of the most common species of cockroaches worldwide. They usually reside in human habitats, where they act as hosts for parasites, viruses, bacteria and pathogenic fungi and can cause severe allergic reactions in humans [29–32]. As these insects are difficult to eradicate, due to their high rates of reproduction and resistance to commonly-used pesticides, biological control strategies based on the use of entomopathogenic fungi are becoming an increasingly desirable option [33–35].

The aim of the present work was to identify any relationships between the cuticular FFA profiles of two cockroach species, *B*. *orientalis* and *B*. *germanica*, and the efficiency of fungal enzymes in hydrolysing the insect cuticle.

## Results

### Susceptibility of cockroaches to fungal infection

Exposure of *B*. *orientalis* and *B*. *germanica* imagines and oothecae to sporulating *C*. *coronatus* colonies showed high resistance of both cockroach species to fungal infection. No infection or mortality was observed in either the control or fungus-threated groups of *B*. *orientalis*. Mortality of fungus treated *B*. *germanica* was very low and comparable to the control groups (Table 1 and S1 Table).

**Table 1:** The susceptibility of *B*. *orientalis* and *B*. *germanica* to fungal infection.

Insect treatment		Tested object	N	Mortality [%±SD]
*B*. *orientalis*	control	imago	20	0 ± 0
		ootheca	10*	0 ± 0**
	fungal infection	imago	30	0 ± 0
		ootheca	30*	0 ± 0**
*B*. *germanica*	control	imago	25	4 ± 8
		ootheca	25*	4 ± 4**
	fungal infection	imago	30	3 ± 8
		ootheca	25*	8 ± 5**

The insects were exposed to sporulating *C*. *coronatus* colonies as described in Materials and methods. The susceptibility to fungal infection is expressed as percentage of mortality in tested populations.

* the number of tested oothecae each containing on average 16 eggs (*B*. *orientalis*) and 40 eggs (*B*. *germanica*), respectively.

** the total percentage of larvae which died during the three days after hatching from oothecae. Percentage of larvae hatching from control and fungus exposed oothecae was 100% in both species (for raw data see supplementary S1 Table).

### Enzyme activity in cell-free *C*. *coronatus* filtrate

The proteolytic, chitinolytic and lipolytic activities of the cell-free *C*. *coronatus* filtrate of were measured as described in the Materials and methods section. The highest activity was demonstrated by elastase (55.31±21.83 mM/min/mg protein): 24-times higher than NAGase (2.32±1.55 mM/min/mg protein; *P* = 0.0028, *F*_(3,6)_ = 196.60), 553-times higher than chitobiosidase (0.10±0.04 mM/min/mg protein; *P* = 0.0023, *F*_(3,6)_ = 258083.00), and 1844-times higher than lipase (0.03±0.001 mM/min/mg protein; *P* = 0.0023, *F*_(3,6)_ = 3411192.00).

### Hydrolysis of cuticular proteins by *C*.* coronatus* enzymes

**Fig 1:**
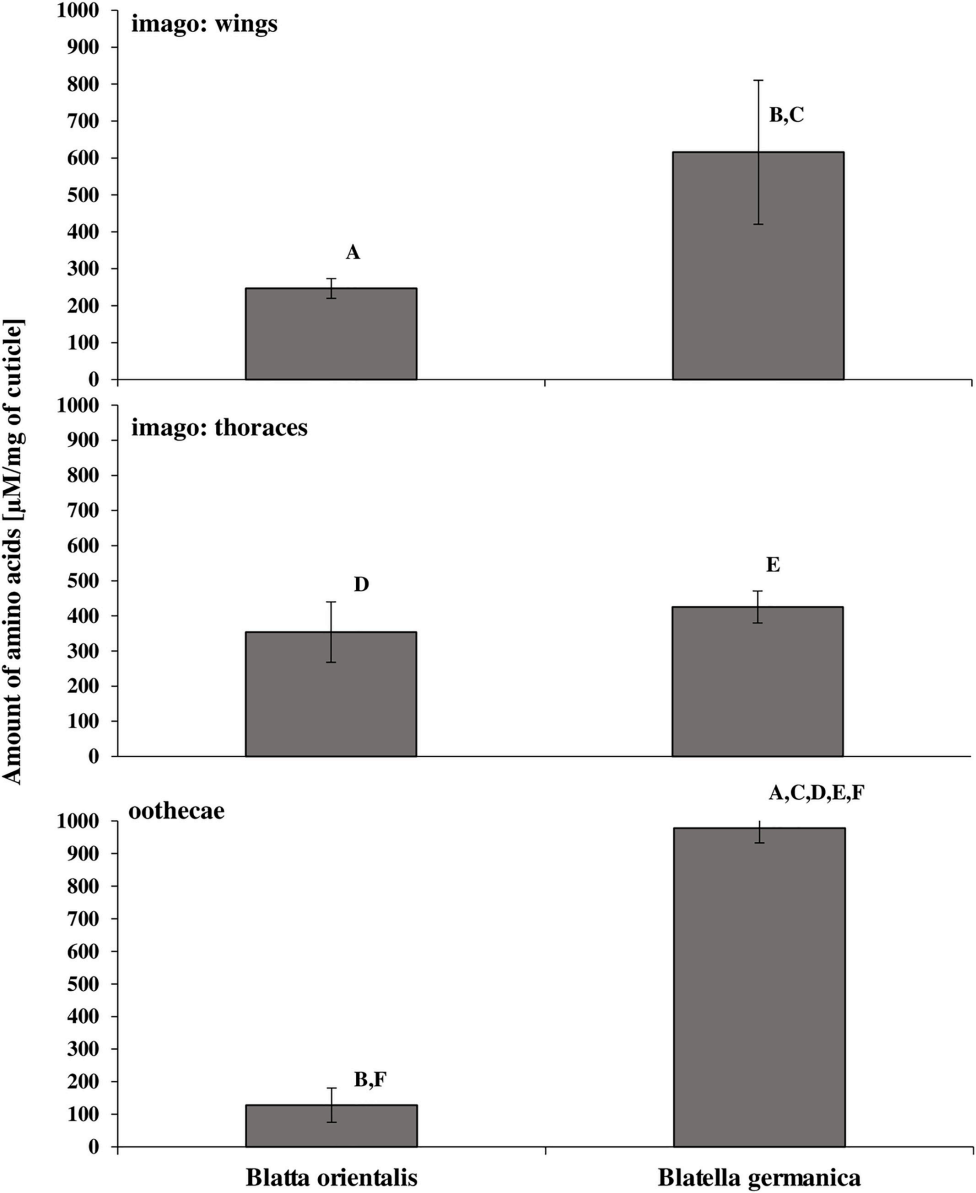
Hydrolysis of cuticular protein by *C*. *coronatus* proteases. Amino acid released during eight hours of incubation is presented as mean ± standard deviation μm/mg of cuticle from wings, thoraces and oothecae of the two cockroach species. Statistically significant differences are marked with the same letter (ANOVA, Tukey's HSD test, p<0.05, for raw data see S2 Table: protein).

The effectiveness of fungal proteolytic enzymes in the culture medium during the 3 weeks of the *C*. *coronatus in vitro* cultivation was measured as the amounts of amino acids released from insect cuticle preparations. The greatest amounts of amino acids were produced during enzymatic digestion of *B*. *germanica* oothecae (978.29±45.49 μM/mg cuticle), and the least (127.86±52.69 μM/mg cuticle F_(5, 12)_ = 14.37, p = 0.0001) for *B*. *orientalis* oothecae. Higher concentrations of amino acids were released from the thoraces, wings and imago of *B*. *germanica* than *B*. *orientalis*. Also 7.7-times more amino acids were released from *B*. *germanica* oothecae than *B*. *orientalis*. Results are given in Fig 1 and supplementary S2 Table.

### Hydrolysis of cuticular chitin by *C*.* coronatus* enzymes

**Fig 2:**
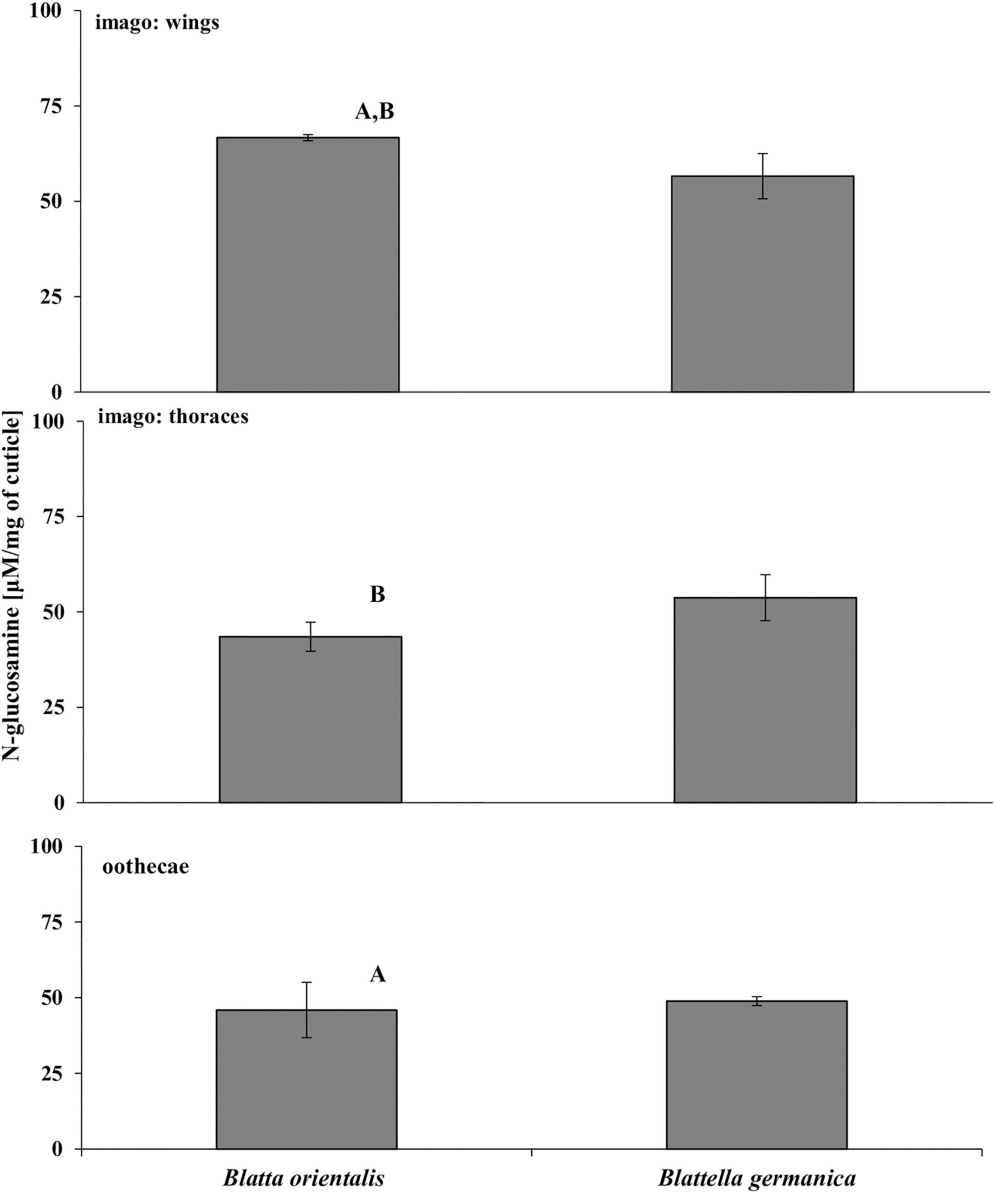
Hydrolysis of cuticular chitin by *C*. *coronatus* chitinases. *N*-glucosamine released during eight hours of incubation is presented as mean ± standard deviation μm/mg of cuticle from wings, thoraces and oothecae of the two cockroach species. Statistically significant differences are marked with the same letter (ANOVA, Tukey's HSD test, p<0.05, for raw data see S2 Table: chitin).

The effectiveness of hydrolysis by the *C*. *coronatus* chitinolytic enzymes was found to be similar in all samples, measured as the concentration of *N-*glucosamine (Fig 2 and S2 Table). The highest levels of *N*-glucosamine were observed for *B*. *orientalis* wings (66.70±0.80 μM/mg cuticle), and the lowest (43.49±3.80 μM/mg cuticle; F_(5,12)_ = 4.98, p = 0.0106) for *B*. *orientalis* thoraces.

### Hydrolysis of cuticular lipids by *C*.* coronatus* enzymes

**Fig 3:**
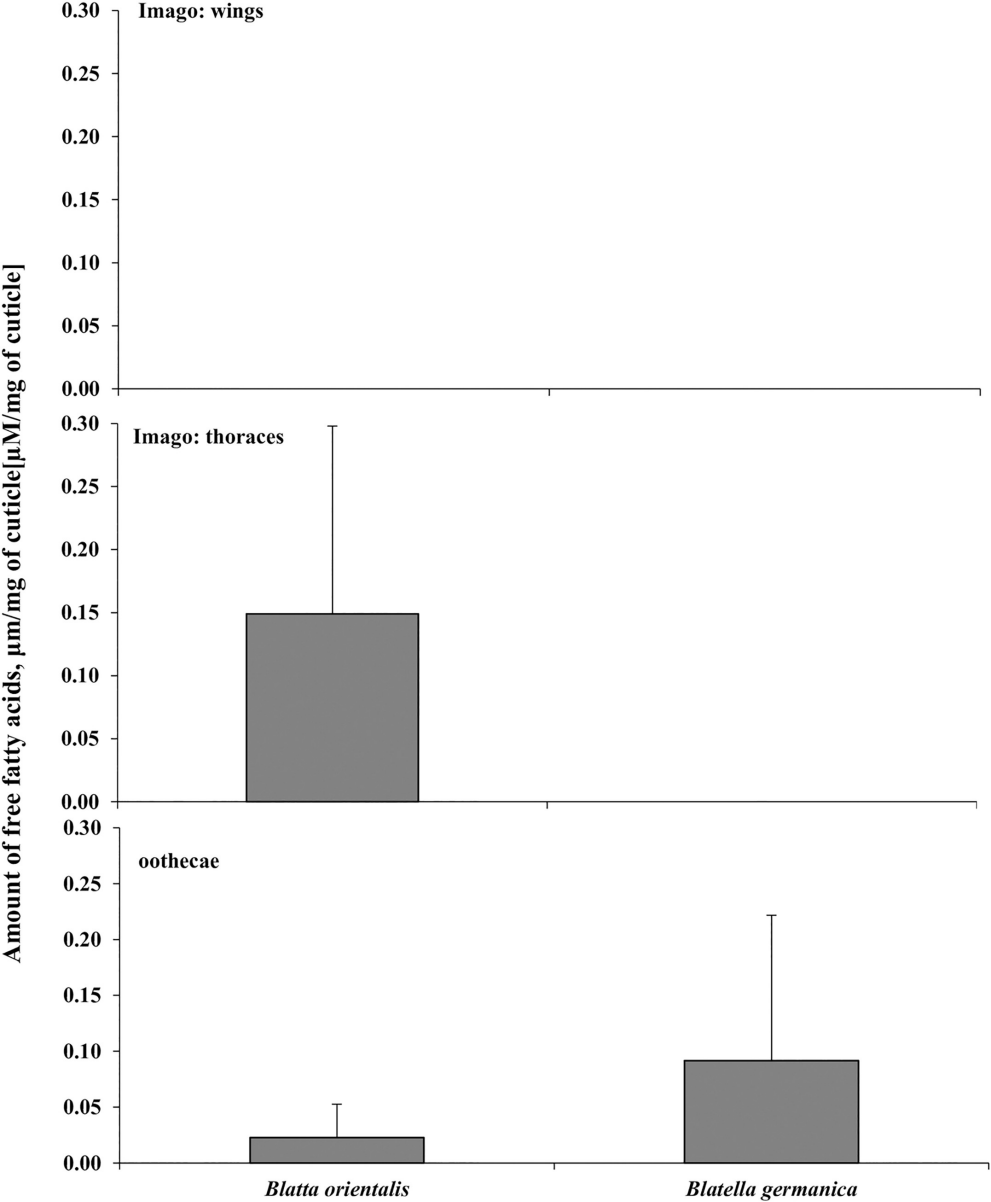
Hydrolysis of cuticular lipids by *C*. *coronatus* lipases. Free fatty acids released during eight hours of incubation are presented as mean ± standard deviation μm/mg of cuticle from wings, thoraces and oothecae of the two cockroach species (for raw data see S2 Table: lipid).

The release of free fatty acids, indicating lipolytic activity, was observed only after digestion of *B*. *germanica* oothecae (0.09±0.13 μM/mg cuticle), *B*. *orientalis* oothecae (0.02±0.03 μM/mg cuticle) and *B*. *orientalis* thoraces (0.15±0.16 μM/mg cuticle) (Fig 3 and S3 Table). These differences were not statistically significant (F_(5, 12) =_ 1.14, p = 0.3918).

### GC-MS analyses of cuticular FFAs

In almost all cases, significantly higher cuticular FFAs were extracted from *B*. *germanica* than *B*. *orientalis*: whole body extracts: 2.6 vs. 1.1 mg/g body; wings extracts: 52.8 vs. 11.4 mg/g wing; thoraces extracts: 30.1 vs. 1.8 mg/g thorax (Table 2). However, for the oothecae, FFA concentrations were 6.3 mg/g for *B*. *germanica* vs. 8.1 mg/g for *B*. *orientalis*. In both species, the highest levels of substances were found in the wings and thoraces.

**Table 2:** Numbers of *B*. *orientalis* and *B*. *germanica* used here and mass of the extracts.

Extracts made from:	N	Insect/body part mass (g)	Extract mass (mg)		
			I	II	III
*B*. *orientalis*					
whole insects	12	5.98	5.61	0.95	5,40
oothecae	80	1.24	5.49	4.60	4.77
wings	539	0.88	6.84	3.20	3.21
thoraces	274	1.26	1.21	1.11	2.26
*B*. *germanica*					
whole insects	100	6.32	14.22	2.54	36.25
oothecae	50	1.69	5.38	5.27	6.60
wings	377	0.27	9.25	5.00	9.67
thoraces	186	0.07	1.86	0.25	0.75

N–total number of individuals; I–petroleum ether extract; II–dichloromethane extract; III–dichloromethane extract after sonification

Both species yielded low FFA content in whole body extracts: 5.46±0.70 μg/g b.w. for *B*. *orientalis* and 5.42±0.65μg/g b.w. for *B*. *germanica*. These differences increased for individual body parts (Table 3 and S3 Table).

**Table 3:** Fatty acid contents in the cuticular lipids (sum of I and II extracts) extracted from the whole adults, oothecae, wings and thoraces of *B*. *orientalis* and *B*. *germanica* (μg/g of insect body or structure) and their antifungal activity.

FFA	Antifungal activity*	adults		oothecae		wings		thoraces	
		*B*. *orientalis*	*B*. *germanica*	*B*. *orientalis*	*B*. *germanica*	*B*. *orientalis*	*B*. *germanica*	*B*. *orientalis*	*B*. *germanica*
Butanoic acid C4:0	NDT	NDC ^A^	0.04±0.02 ^A,B,C,DE,F,G^	NDC ^B^	NDC ^C^	NDC ^D^	NDC ^E^	NDC ^F^	NDC ^G^
Pentanoic acid C5:0	NDT	NDC ^A^	0.04±0.00 ^A,B,C,D,E,F,G^	NDC ^B^	NDC ^C^	NDC ^D^	NDC ^E^	NDC ^F^	NDC ^G^
Hexanoic acid C6:0	YES	0.10±0.04 ^A,F^	0.43±0.56 ^B,G^	0.36±0.12 ^C,H^	0.88±0.07 ^D,I^	4.32±0.16 ^A,B,C,D,E^	14.58±0.36 ^A,B,C,D,J^	0.63±0.05 ^E,J^	13.93±0.60 ^E,F,G,H,I^
Heptanoic acid C7:0	NDT	NDC ^A,B,C^	NDC ^D,E,F^	0.20±0.07 ^A,E,O,G,H^	0.14±0.01 ^B,F,I,J,K^	NDC ^O,I,L^	NDC ^G,J,M^	NDC ^H,K,N^	0.12±0.06 ^C,D,L,M,N^
Octanoic acid C8:0	NDT	0.11±0.01 ^A,C^	0.14±0.01 ^B,D^	0.56±0.19 ^E,F^	0.47±0.02 ^G,H^	2.07±0.06 ^A,B,C,D,E,F,G,H,I^	7.32±0.93 ^A,B,E,G,I^	0.35±0.00 ^I,R^	6.24±0.86 ^C,D,F,H,O,R^
Nonanoic acid C9:0	NDT	0.29±0.01 ^A^	0.23±0.02 ^B^	1.01±0.37 ^C^	0.82±0.05 ^D^	3.84±0.24 ^A,B,C,D,E^	15.12±0.77 ^A,B,C,D,E,^	0.40±0.04 ^E^	17.18±1.06 ^A,B,C,D,E^
Decanoic acid C10:0	NDT	0.05±0.04 ^A^	0.03±0.01 ^B^	0.09±0.02 ^C^	0.08±0.01 ^D^	0.19±0.08 ^E^	NDC ^F^	0.03±0.01 ^G^	3.30±2.90 ^A,B,C,D,E,F,G^
Dodecanoic acid C12:0	NDT	0.19±0.00 ^A^	0.10±0.01 ^B^	0.74±0.02 ^C^	0.29±0.03 ^D^	1.65±0.23 ^A,B,C,D,E^	12.66±0.63 ^A,B,C,D,E^	0.29±0.02 ^E^	13.89±0.37 ^A,B,C,D,E^
Tridecanoic acid C13:0	YES	NDC ^A^	NDC ^B^	0.18±0.00 ^A,B,C,D,E,F,G^	NDC ^C^	NDC ^D^	NDC ^E^	NDC ^F^	NDC ^G^
Tetradecenoic acid C14:1	YES	NDC ^A^	NDC ^B^	NDC ^C^	0.02±0.00 ^A,B,C,D,E,F,G^	NDC ^D^	NDC ^E^	NDC ^F^	NDC ^G^
Tetradecanoic acid C14:0	NDT	0.15±0.03 ^A,B^	0.09±0.01 ^C,D^	3.74±0.34 ^A,C,E,F^	0.28±0.01 ^E,G,H^	3.65±0.18 ^B,D,G,I,^	71.18±1.28 ^A,C,G,J^	0.78±0.02 ^F,I,J^	85.30±0.78 ^A,C,H,I,^
Pentadecenoic acid C15:1	NDT	NDC ^A^	NDC ^B^	NDC ^C^	0.04±0.00 ^A,B,C,D,E,F,G^	NDC ^D^	NDC ^E^	NDC ^F^	NDC ^G^
Pentadecanoic acid C15:0	SLIGHT	0.03±0.00 ^A,B^	0.03±0.01 ^C,D^	1.70±0.11 ^A,C,E,F^	0.10±0.01 ^E,G,H^	1.77±0.12 ^B,D,G,I^	8.08±0.22 ^A,C,G,J^	0.32±0.01 ^F,I,J^	9.48±0.34 ^A,C,H,I^
Hexadecenoic acid C16:1	NDT	NDC ^A^	0.06±0.03 ^B^	6.43±1.68 ^C^	0.50±0.04 ^D^	2.89±0.20 ^E^	297.38±4.77 ^A,B,C,D,E,J^	0.98±0.02 ^J^	331.30±7.27 ^A,B,C,D,E,J^
Hexadecanoic acid C16:0	NDT	2.17±0.22 ^A,B^	1.30±0.73 ^C,D^	106.74±12.43 ^A,C,E,F^	7.41±0.51 ^E,G^	96.01±20.83 ^B,D,G,H^	2888.11±20.58 ^A,C,G,H^	16.75±0.05 ^F,H^	3229.99±43.68 ^A,C,G,H^
Heptadecenoic acid C17:1	SLIGHT	NDC ^A^	NDC ^B^	1.27±0.15 ^C^	0.04±0.01 ^D^	0.95±0.11 ^E^	14.28±1.22 ^A,B,C,D,E,J^	0.45±0.02 ^J^	20.93±0.99 ^A,B,C,D,E,J^
Heptadecanoic acid C17:0	NO	0.03±0.01 ^A,B^	0.47±0.74 ^D^	1.57±0.13 ^C^	0.12±0.00 ^F,E^	2.46±0.07 ^A,F^	29.16±1.46 ^B,D,C,E,G^	0.56±0.02 ^G^	36.74±1.18 ^B,D,C,E,G^
Octadecatrienoic acid C18:3	SLIGHT	NDC ^A^	NDC ^B^	9.10±1.66 ^A,B,C,D,E,F^	2.12±0.25 ^A,B,C,D,E,F^	NDC ^C^	NDC ^D^	NDC ^E^	NDC ^F^
Octadecadienoic acid C18:2	NDT	0.38±0.11 ^A^	0.50±0.01 ^B^	78.74±20.91 ^C^	1.07±0.02 ^A,D^	120.56±1.44 ^A,B,E^	1700.88±62.15 ^A,B,C,D,E^	30.43±0.53 ^E^	2437.16±51.51 ^A,B,C,D,E^
Octadecenoic acid C18:1	NDT	0.56±0.03 ^A^	0.95±0.04 ^B^	211.05±52.85 ^A,B,C,D^	3.89±0.20 ^C,E^	195.24±2.34 ^A,B,E,F^	4542.11±52.74 ^A,B,D,E^	38.29±1.71 ^D,F^	5300.31±45.7 ^A,B,C,F^
Octadecanoic acid C18:0	NDT	0.80±0.13 ^A^	0.90±0.07 ^B^	44.18±11.20 ^A,B,C,D^	NDC ^C^	79.08±14.47 ^A,B,C,D^	808.21±12.00 ^A,B,C,D^	12.32±0.12 ^D^	863.89±13.33 ^A,B,C,D^
Nonadecenoic acid C19:1	SLIGHT	NDC ^A^	NDC ^B^	NDC ^C^	NDC ^D^	NDC ^E^	NDC ^F^	0.56±0.07 ^A,B,C,D,E,F,G^	NDC ^G^
Nonadecanoic acid C19:0	NDT	NDC ^A^	NDC ^B^	0.46±0.09 ^C^	NDC ^D^	NDC ^E^	NDC ^F^	NDC ^G^	4.79±1.36 ^A,B,C,D,E,F,G^
Eicosatetraenoic acid C20:4	YES	NDC ^A^	NDC ^B^	NDC ^C^	NDC ^D^	4.80±0.19 ^E^	116.51±4.00 ^A,B,C,D,E,F^	2.38±0.06 ^F^	208.87±5.99 ^A,B,C,D,E,F^
Eicosatrienoic acid C20:3	SLIGHT	NDC ^A^	NDC ^B^	0.51±0.86 ^C^	NDC ^D^	5.14±0.12 ^A,B,C,D,E^	10.02±1.52 ^A,B,C,D,E^	0.35±0.13 ^E^	28.01±1.67 ^A,B,C,D,E^
Eicosadienoic acid C20:2	SLIGHT	NDC ^A^	NDC ^B^	NDC ^C^	NDC ^D^	NDC ^E^	8.73±5.73 ^A,B,C,D,E,F^	NDC ^F^	7.53±6.55
Eicosenoic acid C20:1	SLIGHT	NDC ^A^	NDC ^B^	NDC ^C^	NDC ^D^	NDC ^E^	36.99±4.61 ^A,B,C,D,E,F^	NDC ^F^	18.83±2.17 ^A,B,C,D,E,F^
Eicosanoic acid C20:0	NO	NDC ^A^	0.03±0.00 ^B^	1.78±0.24 ^A,B,C,D^	0.25±0.01 ^C^	4.35±0.41 ^A,B,C,D^	14.11±0.37 ^A,B,C,D^	0.50±0.03 ^D^	15.23±0.44 ^A,B,C,D^
Docosanoic acid C22:0	NDT	0.13±0.06 ^A^	0.04±0.00 ^B^	2.43±1.42 ^A,B,C,E,F^	0.46±0.01 ^C^	1.44±0.20 ^D^	NDC ^E^	0.33±0.11 ^F^	11.39±0.50 ^A,B,C,D, E,F^
Tetracosanoic acid C24:0	NO	0.23±0.10 ^A,G^	0.05±0.00 ^B,H^	5.08±1.31 ^A,B,C,D,E,F^	0.43±0.02 ^C,I^	NDC ^D,J^	NDC ^E,K^	NDC ^F,L^	6.26±0.58 ^G,H,I,J,K,L^
Hexacosanoic acid C26:0	NO	0.24±0.06 ^A^	NDC ^B^	2.82±1.62 ^A,B,C,D,E,F^	0.38±0.02 ^C^	NDC ^D^	NDC ^E^	NDC ^F^	5.15±0.75 ^A,B,C,D,E,F^
Sum of FFA		5.46±0.70 ^A,B,^	5.42±0.65 ^C,D^	481.80±134.56 ^A,C,E,F^	19.89±1.02 ^E,G^	530.40±34.94 ^B,D,G,H^	10595.44±7.97 ^A,B,C,D,G,H^	106.71±1.28 ^F,H^	12675.83±45.53 ^A,B,C,D,G,H^

FFA- free fatty acids. SD—standard deviation; Extract I- petroleum ether extract; Extract II- dichloromethane extracts; NDT–not determined; NDC–not detected; statistically significant differences are marked with the same letters (ANOVA, Test HSD Tukey, p<0.05), for raw data see supplementary S3 Table

*data from [18,36]. Tested fungal species: *Aspergillus niger*, *Beauveria bassiana*, *Candida albicans*, *Candida lipolytica*, *Candida tropicalis*, *Lecanicillium lecanii*, *Metarhizium anisopliae*, *Paecilomyces fumosoroseus*, *Peacilomyces lilacinus*

The highest total FFA level was observed in *B*. *germanica* wings (10595.44±7.97 μg/g) and thoraces (12675.83±45.53 μg/g). In contrast, 20 and 120-times lower FFA content was found in *B*. *orientalis* wings (530.40±34.94 μg/g) and thoraces (106.71±1.28 μg/g) (F_(7,16)_ = 31770.00, p<0.0001). Regarding the oothecae, the total FFA content of *B*. *orientalis* (481.80±134.56 μg/g) was 24 times higher than *B*. *germanica* (19.89±1.02 μg/g).

**Fig 4:**
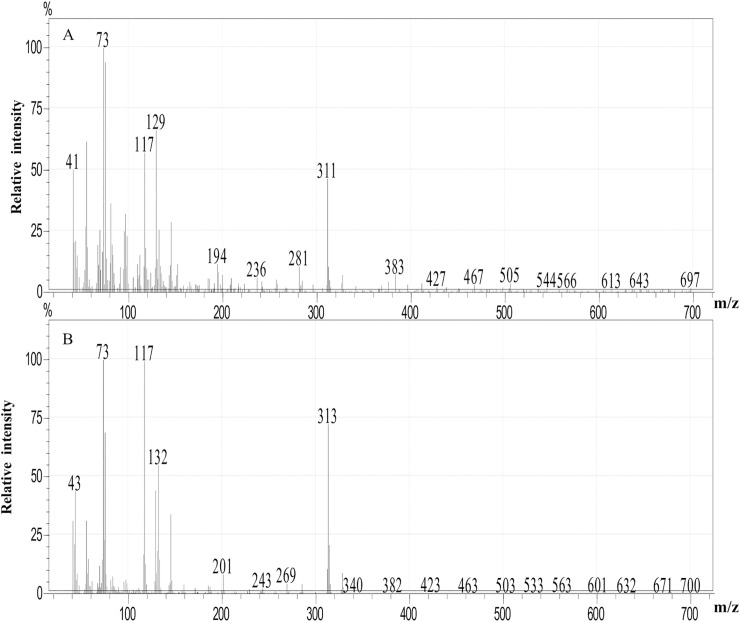
Mass spectra of the trimethylsilyl (TMS) ester of hexadecenoic acid (A) and hexadecanoic acid (B).

Individual FFAs present in each extract were identified and quantified. Example mass spectra of the trimethylsilyl (TMS) esters of hexadecenoic acid (C16:0) and hexadecenoic acid (C16:1) are given in Fig 4.

In the cuticular extracts of adult *B*. *orientalis*, fifteen FFAs were detected: thirteen saturated (C6:0, C8:0, C9:0, C10:0, C12:0, C14:0, C15:0, C16:0, C17:0, C18:0, C22:0, C24:0, C26:0), and two unsaturated (C18:2 and C18:1). In the oothecae, twenty three acids were found: seventeen saturated (C6:0, C7:0, C8:0, C9:0, C10:0, C12:0, C13:0, C14:0, C15:0, C16:0, C17:0, C18:0, C19:0, C20:0, C22:0, C24:0. C26:0) and six unsaturated (C16:1, C17:1, C18:2, C18:1, C18:3, C20:3). Eighteen were demonstrated in wings cuticle: twelve saturated (C6:0, C8:0, C9:0, C10:0, C12:0, C14:0, C15:0, C16:0, C17:0, C18:0, C20:0, C22:0) and six unsaturated (C16:1, C17:1, C18:2, C18:1, C20:4, C20:3). Nineteen FFAs were found in the thoraces extract: 12 saturated (C6:0, C8:0, C9:0, C10:0, C12:0, C14:0, C15:0, C16:0, C17:0, C18:0, C20:0, C22:0) and seven unsaturated (C16:1, C17:1, C18:2, C18:1, C19:1, C20:4, C20:3). Of these C7:0, C13:0, C18:3, and C19:0 were detected only in the oothecae, while C16:1, C17:1, C20:3 and C20:0 were absent from the whole *B*. *orientalis* adults cuticular extract but were found in extracts from their oothecae, wings and thoraces.

Eighteen FFAs were found in cuticular extracts from *B*. *germanica* adults (whole body): fifteen saturated (C4:0, C5:0, C6:0, C8:0, C9:0 C10:0, C12:0, C14:0, C15:0, C16:0, C17:0, C18:0, C20:0, C22:0, C24:0) and three unsaturated (C16:1, C18:2, C18:1). C4:0, C5:0, C16:1, C20:0 were absent in analogical extracts from *B*. *orientalis*, while C26:0 present in *B*. *orientalis* was absent from *B*. *germanica*. Twenty-one FFAs were found in *B*. *germanica* oothecae: seventeen saturated (C6:0, C7:0, C8:0, C9:0, C10:0, C12:0, C14:0, C15:0, C16:0, C17:0, C20:0, C22:0, C24:0, C26:0) and seven unsaturated (C14:1, C15:1, C16:1, C17:1, C18:2, C18:1, C18:3). C14:1, C15:1 and C18:3 were found only in the oothecae while C7:0 and C26:0 were found in both oothecae and thoraces. Comparing the oothecae of the two species, C14:1 and C15:1 were found only in *B*. *germanica* while C13:0, C18:0, C19:0 and C20:3 were only present in *B*. *orientalis*. Most oothecae FFAs common to both species were present in significantly different amounts (Table 3). Eighteen FFAs were found on the wings cuticle: ten saturated (C6:0, C8:0, C9:0, C12:0, C14:0, C15:0, C16:0, C17:0, C18:0, C20:0) and eight unsaturated (C16:1, C17:1, C18:2, C18:1, C20:4, C20:3, C20:2, C20:1). Interestingly, the C10:0 missing in *B*. *germanica* wings was detected in other *B*. *germanica* extracts, as well as in *B*. *orientalis* wings. C20:1, C20:2 and C22:0 were absent in *B*. *orientalis* but present in *B*. *germanica*. All shared FFAs were present in significantly higher amounts in *B*. *germanica*, ranging from 1.9 (C20:3) to 102.9 times (C16:1). Twenty-four FFAs were detected in the *B*. *germanica* thoraces: sixteen saturated (C6:0, C7:0, C8:0, C9:0, C10:0, C12:0, C14:0, C15:0, C16:0, C17:0, C18:0, C19:0, C20:0, C22:0, C24:0, C26:0) and eight unsaturated (C16:1, C17:1, C18:2, C18:1, C20:4, C20:3, C20:2, C20:1). C19:1 was present only in *B*. *orientalis* thoraces but no other extract. C7:0, C19:0, C20:1, C20:2, C24:0 and C26:0 were absent from *B*. *orientalis* but present in *B*. *germanica* thoraces. All common FFAs were significantly more abundant in *B*. *germanica*: 17.8 (C8:0) to 338 times (C16:1).

Four FFAs predominated in all cuticular extracts: C16:0, C18:2, C18:1 and C18:0 (Table 3). C16:0 predominated in whole insect extracts in both species (2.17±0.22 μg/g bw *B*. *orientalis*; 1.30±0.73 μg/g bw *B*. *germanica*). C16:0 was strong in *B*. *germanica* wings (2888.11±20.58 μg/g wings) and thoraces (3229.99±43.68 μg/g thoraces). C18:1 predominated in *B*. *orientalis* oothecae, (211.05±52.85 μg/g), wings (195.24±2.34 μg/g) and thoraces (38.29±1.71 μg/g), as well as in *B*. *germanica* wings (4542.11±52.74 μg/g) and thoraces (5300.31±45.7 μg/g thoraces). The wings and thoraces of *B*. *germanica* also demonstrated high concentrations of C18:0 (808.21±12.00 μg/g wings, 863.89±13.33 μg/g thoraces) and C18:2 (1700.88±62.15 μg/g wings, 2437.16±51.51 μg/g thoraces).

### GC-MS analyses of internal FFAs

In almost all cases, significantly higher amounts were extracted from *B*. *germanica* than from *B*. *orientalis*. The yields were obtained from *B*. *germanica* whole body (61.51±3.53 μg/g) were17-times greater than for *B*. *orientalis* (3.62±0.17 μg/g) (Table 4 and S3 Table). A similar situation was observed for the wings (197.34±1.80 μg/g *B*. *orientalis* vs. 2291.14±31.31 μg/g *B*. *germanica*) and thoraces (266.96±1.44 μg/g *B*. *orientalis* vs. 1570.31±11.94 μg/g *B*. *germanica*); the opposite was found for oothecae (163.24±23.69 μg/g *B*. *orientalis* vs. 41.21±5.31 μg/g *B*. *germanica*) (Table 4). Fifteen FFAs were detected in *B*. *orientalis* adult whole body extracts: thirteen saturated (C6:0, C8:0, C9:0, C10:0, C12:0, C14:0, C16:0, C17:0, C18:0, C20:0, C22:0, C24:0, C26:0) and two unsaturated (C18:2, C18:1). Similarly, fifteen FFAs were found in *B*. *germanica* adults, but twelve were saturated (C5:0, C6:0, C7:0, C8:0, C9:0 C10:0, C12:0, C14:0, C15:0, C16:0, C17:0, C18:0) and three were unsaturated (C16:1, C18:2, C18:1). C5:0 was found only in the body *of B*. *germanica*, not in any other analysed internal lipid fraction. Most shared FFAs were more abundant in adult *B*. *germanica* than *B*. *orientalis*.

**Table 4:** Fatty acid contents in the internal lipids (extract III) of the adults, oothecae, wings and thoraces of *B*. *orientalis* and *B*. *germanica* (μg/g of insect body or structure) and their antifungal activity.

FFA	Antifungal activity*	adults		oothecae		wings		thoraces	
		*B*. *orientalis*	*B*. *germanica*	*B*. *orientalis*	*B*. *germanica*	*B*. *orientalis*	*B*. *germanica*	*B*. *orientalis*	*B*. *germanica*
Butanoic acid C4:0	NDT	NDC	NDC	NDC	NDC	NDC	NDC	NDC	NDC
Pentanoic acid C5:0	NDT	NDC ^A^	0.18±0.06 ^A,B,C,D,E,F^	NDC ^B^	NDC ^C^	NDC ^D^	NDC ^E^	NDC ^E^	NDC ^F^
Hexanoic acid C6:0	YES	0.07±0.02 ^A,B^	0.94±0.38 ^A,C,^	0.33±0.13 ^D^	0.49±0.04 ^E^	1.41±0.09 ^B,D,E,F^	10.82±0.49 ^B,C,D,E,F^	0.66±0.04 ^F^	6.81±0.29 ^B,C,D,E,F^
Heptanoic acid C7:0	NDT	NDC ^A^	0.18±0.04 ^B^	0.06±0.01 ^C^	0.08±0.03 ^D^	NDC ^E^	NDC ^F^	NDC ^G^	0.81±0.39 ^A,B,C,D,E,F,G^
Octanoic acid C8:0	NDT	0.05±0.03 ^A^	0.31±0.05 ^B^	0.20±0.06 ^C^	0.18±0.05 ^D^	0.61±0.02 ^E^	3.32±0.2 ^A,B,C,D,E,F^	0.38±0.01 ^F^	2.26±0.31 ^A,B,C,D,E,F^
Nonanoic acid C9:0	NDT	0.08±0.02 ^A,B,C^	1.06±0.08 ^A^	0.42±0.06 ^D^	0.75±0.09 ^B^	0.87±0.06 ^C^	8.20±0.50 ^A,B,C,D,E^	0.50±0.03 ^E^	4.15±0.32 ^A,B, C,D,E^
Decanoic acid C10:0	NDT	0.02±0.00	0.08±0.07	0.09±0.03	0.05±0.01	NDC	NDC	0.09±0.04	NDC
Dodecanoic acid C12:0	NDT	0.12±0.02 ^A^	0.30±0.01 ^B,C^	0.35±0.03 ^D,E^	0.23±0.03 ^F,G^	0.54±0.08 ^A^	2.09±0.34 ^A,B,D,F,H,L^	0.34±0.00 ^H,I^	2.27±0.13 ^A,C,E,G,I,M^
Tridecanoic acid C13:0	YES	NDC	NDC	NDC	NDC	NDC	NDC	NDC	NDC
Tetradecenoic acid C14:1	YES	NDC	NDC	NDC	NDC	NDC	NDC	NDC	NDC
Tetradecanoic acid C14:0	NDT	0.04±0.01 ^A,B,C,D^	0.80±0.02 ^A^	1.17±0.19 ^B^	0.40±0.08 ^B,E,F^	1.41±0.11 ^C,E^	12.11±0.22 ^A,B,C,D^	1.40±0.06 ^D,F^	6.31±0.43 ^A, B,C,D^
Pentadecenoic acid C15:1	NDT	NDC	NDC	NDC	NDC	NDC	NDC	NDC	NDC
Pentadecanoic acid C15:0	SLIGHT	NDC ^A,B^	0.09±0.01 ^C,D^	0.45±0.06 ^E,F^	0.11±0.01 ^G,K^	0.77±0.06 ^A,C,G,L^	2.16±0.39 ^A,C,E,G,M^	0.62±0.04 ^B,M^	1.88±0.32 ^B,D,F,K,L^
Hexadecenoic acid C16:1	NDT	NDC ^A,B,C,D^	1.35±0.10 ^A^	1.88±0.28 ^B^	1.48±0.17 ^C^	1.27±0.04 ^D^	42.52±0.34 ^A,B,C,D^	2.16±0.04 ^A,D^	24.33±0.48 ^A,B,C,D^
Hexadecanoic acid C16:0	NDT	1.12±0.11 ^A,B,C^	25.61±1.32 ^A,D^	41.54±5.23 ^A,E^	16.96±1.98 ^A,E,F^	30.14±0.21 ^B,E^	467.16±2.21 ^A,E,F^	37.47±0.41 ^C,D,F^	273.13±1.15 ^A,E,F^
Heptadecenoic acid C17:1	SLIGHT	NDC ^A,B^	NDC ^C,D^	0.32±0.04 ^E,F^	NDC ^G,H^	0.79±0.08 ^A,C,G^	2.55±0.29 ^A,B,C,E,G^	0.10±0.05 ^B,D,F,H^	1.49±0.41 ^A,C,E,G^
Heptadecanoic acid C17:0	NO	0.03±0.01 ^A,B^	0.22±0.06 ^C,F^	0.56±0.06 ^D^	0.15±0.04 ^E,G^	1.00±0.02 ^A^	5.95±0.67 ^A,C,D,E,H^	1.10±0.02 ^B,F,G,H^	3.28±0.46 ^A,C,D,E,H^
Octadecatrienoic acid C18:3	SLIGHT	NDC ^A,B^	NDC ^C,D^	2.42±0.32 ^A,C,E,F,G,H^	2.68±0.36 ^B,D,I,J,K,L^	NDC ^E,I^	NDC ^F,J^	NDC ^G,K^	NDC ^H,L^
Octadecadienoic acid C18:2	NDT	0.60±0.06 ^A^	8.36±0.39 ^B^	31.32±7.73 ^A,B,C^	4.12±0.65 ^C^	53.85±1.41 ^A,B,C^	503.35±7.27 ^A,B,C^	78.62±0.68 ^A,B,C^	430.98±3.13 ^A,B,C^
Octadecenoic acid C18:1	NDT	0.46±0.03 ^A,B^	14.69±1.11 ^C,D^	61.99±6.85 ^A,C,E^	12.71±1.66 ^E,F^	74.11±2.31 ^B,D^	981.41±27.67 ^A,C,E,F^	103.23±2.27 ^A,C,E,^	651.29±6.23 ^A,C,E,F^
Octadecanoic acid C18:0	NDT	0.36±0.04 ^A,B^	7.33±0.60 ^A,C^	11.86±1.32 ^A,D,E,^	NDC ^C,D,F^	24.78±0.70 ^A,F^	174.09±3.12 ^A,F,G^	26.99±0.,34 ^B,C,E,G,^	75.62±1.95 ^A,F,G^
Nonadecanoic acid C19:0	NDT	NDC ^A^	NDC ^B^	0.17±0.03 ^A,B,C,D,E,F^	NDC ^C^	NDC ^D^	NDC ^E^	0.44±0.05 ^A,B,C,D,E,F^	NDC ^F^
Nonadecenoic acid C19:1	SLIGHT	NDC ^A^	NDC ^B^	NDC ^C^	NDC ^D^	NDC ^E^	NDC ^F^	1.31±0.07 ^A,B,C,D,E,F,G^	NDC ^G^
Eicosatetraenoic acid C20:4	YES	NDC ^A^	NDC ^B^	0.77±0.23 ^C^	NDC ^D^	NDC ^E^	48.75±2.11 ^A,B,C,D,E^	7.03±0.22 ^A,B,C,D,E^	59.13±1.43 ^A,B,C,D,E^
Eicosatrienoic acid C20:3	SLIGHT	NDC ^A,B^	NDC ^C,D^	0.70±0.13 ^E,F^	0.11±0.02 ^G,H^	4.12±0.20 ^A,C,E,G,I^	26.66±2.32 ^A,C,E,G,I,J^	0.98±0.01 ^I,J^	3.95±0.54 ^B,D,F,H,J^
Eicosadienoic acid C20:2	SLIGHT	NDC ^A,B^	NDC ^C,D^	0.59±0.12 ^A,C,E,F,G,H^	NDC ^E,I^	NDC ^F,J^	NDC ^G,K^	0.78±0.24 ^B,D,I,J,K,L^	NDC ^H,L^
Eicosenoic acid C20:1	SLIGHT	NDC	NDC	NDC	NDC	NDC	NDC	NDC	NDC
Eicosanoic acid C20:0	NO	0.07±0.02 ^A,B^	NDC ^C,D^	0.80±0.10 ^A,C,E,F^	0.16±0.02 ^E,N^	1.67±0.09 ^A,C,E,F,G^	NDC ^F,H^	1.20±0.06 ^B,D,N,G,H,I^	2.76±0.34 ^A,C,E,F,I^
Docosanoic acid C22:0	NDT	0.14±0.01 ^A^	NDC ^B^	1.53±0.20 ^A,B,C,D,E,F^	0.26±0.06 ^C^	NDC ^D^	NDC ^E^	0.64±0.15 ^F^	7.23±0.49 ^A,B,C,D,E,F^
Tetracosanoic acid C24:0	NO	0.28±0.01 ^A^	NDC ^B^	2.02±0.24 ^C^	0.25±0.08 ^D^	NDC ^E^	NDC ^F^	NDC ^G^	12.61±1.64 ^A,B,C,D,E,F,G^
Hexacosanoic acid C26:0	NO	0.19±0.04 ^A^	NDC ^B^	1.69±0.24 ^A,B,C,D,E,F,G^	NDC ^C^	NDC ^D^	NDC ^E^	NDC ^F^	NDC ^G^
Sum of FFA		3.62±0.17 ^A,B^	61.51±3.53 ^A,B^	163.24±23.69 ^B,C^	41.21±5.31 ^C,D^	197.34±1.8 ^A,D^	2291.14±31.31 ^A,BC,^	266.96±1.44 ^A,B,C^	1570.31±11.94 ^A,BC^

FFA- free fatty acids; SD—standard deviation; Extract III- dichloromethane extracts after sonification; NDT–not determined; NDC–not detected; statistically significant differences are marked with the same letters (ANOVA, Test HSD Tukey, p<0.05), for raw data see supplementary S3 Table

* data from [18,36] Tested fungal species: *Aspergillus niger*, *Beauveria bassiana*, *Candida albicans*, *Candida lipolytica*, *Candida tropicalis*, *Lecanicillium lecanii*, *Metarhizium anisopliae*, *Paecilomyces fumosoroseus*, *Peacilomyces lilacinus*

A greater variety of FFA types were found in the oothecae of both species. Twenty-four in *B*. *orientalis*, including sixteen saturated (C6:0, C7:0, C8:0, C9:0, C10:0, C12:0, C14:0, C15:0, C16:0, C17:0, C18:0, C19:0, C20:0, C22:0, C24:0, C26:0) and eight unsaturated (C16:1, C17:1, C18:2, C18:1, C18:3, C20:4, C20:3, C20:2) and eighteen in *B*. *germanica*: thirteen saturated (C6:0, C7:0, C8:0, C9:0, C10:0, C12:0, C14:0, C15:0, C16:0, C17:0, C20:0, C22:0, C24:0) and five unsaturated (C16:1, C18:2, C18:1, C18:3, C20:3). C18:3 was found exclusively in the oothecae of both species. Most shared FFAs were more abundant in *B*. *orientalis* (Table 4).

Fifteen FFAs were found in *B*. *orientalis* wings extracts: ten saturated (C6:0, C8:0, C9:0, C12:0, C14:0, C15:0, C16:0, C17:0, C18:0, C20:0) and five unsaturated (C16:1, C17:1, C18:2, C18:1, C20:3). Fifteen were found in *B*. *germanica* wings, but nine were saturated (C6:0, C8:0, C9:0, C12:0, C14:0, C15:0, C16:0, C17:0, C18:0) and six unsaturated (C16:1, C17:1, C18:2, C18:1, C20:4, C20:3). C20:4 was present in *B*. *germanica* wings but not in *B*. *orientalis* wings, and vice versa for C20:0. All shared FFAs were significantly more abundant in *B*. *germanica*: 2.8 (C15:0) to 15.5 times (C16:0).

Twenty-one FFAs were found in *B*. *orientalis* thoraces, thirteen saturated (C6:0, C8:0, C9:0, C10:0, C12:0, C14:0, C15:0, C16:0, C17:0, C18:0, C19:0, C20:0, C22:0) and eight unsaturated (C16:1, C17:1, C18:2, C18:1, C19:1, C20:4, C20:3, C20:2), while 19 FFAs were detected in *B*. *germanica* thoraces: thirteen saturated (C6:0, C7:0, C8:0, C9:0, C12:0, C14:0, C15:0, C16:0, C17:0, C18:0, C20:0, C22:0, C24:0) and six unsaturated (C16:1, C17:1, C18:2, C18:1, C20:4, C20:3). C19:1 was present only in *B*. *germanica* thoraces, and in no other extracts, while C10:0, C19:0, C20:2 and C24:0 were absent in *B*. *germanica* thoraces but present in *B*. *orientalis* thoraces. All shared FFAs were significantly more abundant in *B*. *germanica*: 2.9 (C17:0) to 14.9 times (C17:1).

Four FFAs predominated in all analysed internal extracts: C16:0, C18:2, C18:1 and C18:0 (Table 4). C16:0 was the most abundant FFA in all whole insect extracts (1.12±0.11 μg/g b.w. *B*. *orientalis*; 1325.61±1.32 μg/g b.w. *B*. *germanica*). C16:0 was abundant in *B*. *germanica* wings (467.16±2.21μg/g) and thoraces (273.13±1.15 μg/g), but less so in the oothecae (41.54±1.15 μg/g *B*. *orientalis*; 16.96±1.98 μg/g *B*. *germanica*). The highest concentrations of C18:1 were measured in *B*. *germanica* wings (981.41±27.67 μg/g) and thoraces (651.29±6.23 μg/g), as well as high concentrations of C18:2 (503.35±7.27 μg/g wings, 430.98±3.13 μg/g thoraces) and C18:0 (174.09±3.12 μg/g wings, 75.62±1.95 μg/g thoraces). C16:0, C18:2, C18:1 and C18:0 predominated in *B*. *orientalis* extracts.

*B*. *orientalis* demonstrated higher concentrations of FFAs from the cuticle (except C16:0, C17:0, C18:0, C18:1, C18:2), while in *B*. *germanica* higher FFA levels were detected inside the body. For the oothecae, *B*. *germanica* had a similar profile, while for *B*. *orientalis*, all FFAs, except C14:0 and C20:4, were more abundant in extracts I and II than extract III.

Regarding the wings, higher concentrations of all FFAs were found in the cuticle for both species, apart from C20:3 for *B*. *germanica*. Regarding the thoraces, higher concentrations of FFAs were present in the combined extracts I and II for *B*. *germanica* (except for C7:0 and C24:0), while all FFAs were more abundant in extract III for *B*. *orientalis* (Tables 3 and 4).

### Correlations between rates of cuticle hydrolysis and FFAs profiles

The correlation coefficients obtained between cuticle hydrolysis and FFA profile varied from |0.1| to |1.0| (Tables 5 and 6). Scatterplots indicate both positive and negative linear correlations with various strengths. A correlation between cuticle FFAs concentration and the effectiveness of fungal enzymes was recognized in cases where a strong correlation (*r* ≥0.6 or *r* ≤−0.6) was observed in both cockroach species. Proteolytic degradation of the cuticle was negatively correlated with cuticular C10:0 in oothecae, and C6:0, C9:0, C16:0 and C20:0 in thoraces, and positively correlated with C17:0 in oothecae, C6:0 in wings, C12:0 and C20:3 in thoraces (Table 5). Fungal protease efficiency negatively correlated with internal C15:0, C16:0, C17:0 and C20:3 levels for thoraces, and positively correlated with internal C6:0 and C14 for wings (Table 6). Fungal chitinase efficiency positively correlated with cuticular C12:0, C14:0, C17:1 and C20:3 content in oothecae, C16:1 in wings, and C6:0 and C10:0 in thoraces, and negatively correlated with C18:0 in wings and thoraces, and with C18:2 in wings (Table 5). In thoraces, chitin degradation positively correlated with internal C6:0 and C20:3, and negatively correlated with internal C17:1, C18:2 and C20:4.

**Table 5:** Correlation between the concentration of compounds identified in the cuticle of two cockroach species and the efficiency of *C*. *coronatus* proteases, chitinases and lipases in degrading the cockroach cuticle.

FFA	Effect on *C*. coronatus^*^	Proteases			Chitinases			Lipases		
		oothecae	wings	thoraces	oothecae	wings	thoraces	oothecae	wings	thoraces
C4:0	Negative	BO (NDC)	BO (NDC)	BO (NDC)	BO (NDC)	BO (NDC)	BO (NDC)	BO (NDC)	BO (NDC)	BO (NDC)
		BG (NDC)	BG (NDC)	BG (NDC)	BG (NDC)	BG (NDC)	BG (NDC)	BG (NDC)	BG (NDC)	BG (NDC)
C5:0	Negative	BO (NDC)	BO (NDC)	BO (NDC)	BO (NDC)	BO (NDC)	BO (NDC)	BO (NDC)	BO (NDC)	BO (NDC)
		BG (NDC)	BG (NDC)	BG (NDC)	BG (NDC)	BG (NDC)	BG (NDC)	BG (NDC)	BG (NDC)	BG (NDC)
C6:0	Negative	BO (0.92)	BO (0.79)	BO (-0.90)	BO (0.84)	BO (-0.55)	BO (0.64)	BO (-0.94)	BO (NDC)	BO (0.57)
		BG (-0.98)	BG (0.95)	BG (-0.66)	BG (0.25)	BG (0.60)	BG (0.92)	BG (0.81)	BG (NDC)	BG (NDC)
C7:0	Negative	BO (0.96)	BO (NDC)	BO (NDC)	BO (0.77)	BO (NDC)	BO (NDC)	BO (-0.98)	BO (NDC)	BO (NDC)
		BG (-0.99)	BG (NDC)	BG (-0.28)	BG (-0.07)	BG (NDC)	BG (-0.83)	BG (0.59)	BG (NDC)	BG (NDC)
C8:0	Negative	BO (0.88)	BO (0.96)	BO (1.00)	BO (0.88)	BO (-0.83)	BO (-0.45)	BO (-0.91)	BO (NDC)	BO (-0.37)
		BG (-0.97)	BG (-0.18)	BG (0.17)	BG (-0.18)	BG (0.50)	BG (0.88)	BG (0.49)	BG (NDC)	BG (NDC)
C9:0	Negative	BO (0.98)	BO (0.81)	BO (-0.80)	BO (0.71)	BO (-0.58)	BO (-0.45)	BO (-0.99)	BO (NDC)	BO (-0.53)
		BG (-0.97)	BG (-0.76)	BG (-0.97)	BG (-0.18)	BG (-0.20)	BG (0.54)	BG (0.49)	BG (NDC)	BG (NDC)
C10:0	Negative	BO (-0.88)	BO (0.13)	BO (0.92)	BO (-0.11)	BO (-0.44)	BO (0.99)	BO (0.85)	BO (NDC)	BO (0.98)
		BG (-0.75)	BG (NDC)	BG (-0.33)	BG (-0.61)	BG (NDC)	BG (1.00)	BG (0.05)	BG (NDC)	BG (NDC)
C12:0	Negative	BO (0.93)	BO (-0.51)	BO (0.80)	BO (0.83)	BO (0.20)	BO (-0.81)	BO (-0.95)	BO (NDC)	BO (-0.75)
		BG (0.32)	BG (-0.85)	BG (0.75)	BG (0.92)	BG (-1.00)	BG (-0.86)	BG (0.46)	BG (NDC)	BG (NDC)
C13:0	Negative	BO (0.11)	BO (NDC)	BO (NDC)	BO (-0.76)	BO (NDC)	BO (NDC)	BO (-0.04)	BO (NDC)	BO (NDC)
		BG (NDC)	BG (NDC)	BG (NDC)	BG (NDC)	BG (NDC)	BG (NDC)	BG (NDC)	BG (NDC)	BG (NDC)
C14:1	Negative	BO (NDC)	BO (NDC)	BO (NDC)	BO (NDC)	BO (NDC)	BO (NDC)	BO (NDC)	BO (NDC)	BO (NDC)
		BG (0.28)	BG (NDC)	BG (NDC)	BG (0.94)	BG (NDC)	BG (NDC)	BG (0.50)	BG (NDC)	BG (NDC)
C14:0	Negative	BO (0.79)	BO (0.08)	BO (0.90)	BO (0.95)	BO (-0.39)	BO (1.00)	BO (-0.83)	BO (NDC)	BO (0.19)
		BG (-0.73)	BG (-0.65)	BG (-0.96)	BG (0.73)	BG (-1.00)	BG (0.56)	BG (1.00)	BG (NDC)	BG (NDC)
C15:1	Negative	BO (NDC)	BO (NDC)	BO (NDC)	BO (NDC)	BO (NDC)	BO (NDC)	BO (NDC)	BO (NDC)	BO (NDC)
		BG (-0.05)	BG (NDC)	BG (NDC)	BG (1.00)	BG (NDC)	BG (NDC)	BG (0.76)	BG (NDC)	BG (NDC)
C15:0	Positive	BO (-0.46)	BO (0.10)	BO (0.40)	BO (0.47)	BO (-0.42)	BO (-0.98)	BO (0.40)	BO (NDC)	BO (-0.96)
		BG (-0.93)	BG (-1.00)	BG (0.06)	BG (-0.42)	BG (-0.80)	BG (0.93)	BG (-0.90)	BG (NDC)	BG (NDC)
C16:1	Negative	BO (0.92)	BO (-0.92)	BO (-0.10)	BO (0.84)	BO (1.00)	BO (0.99)	BO (-0.95)	BO (NDC)	BO (1.00)
		BG (-0.93)	BG (0.73)	BG (-0.98)	BG (0.43)	BG (1.00)	BG (0.51)	BG (0.91)	BG (NDC)	BG (NDC)
C16:0	Negative	BO (0.91)	BO (0.28)	BO (-0.90)	BO (0.86)	BO (-0.57)	BO (-0.15)	BO (-0.94)	BO (NDC)	BO (-0.24)
		BG (-0.70)	BG (0.98)	BG (-0.99)	BG (-0.66)	BG (0.90)	BG (0.46)	BG (-0.03)	BG (NDC)	BG (NDC)
C17:1	Negative	BO (0.97)	BO (0.19)	BO (-0.70)	BO (0.75)	BO (-0.49)	BO (0.82)	BO (-0.98)	BO (NDC)	BO (0.76)
		BG (0.28)	BG (0.35)	BG (0.89)	BG (0.94)	BG (0.90)	BG (-0.71)	BG (0.50)	BG (NDC)	BG (NDC)
C17:0	Negative	BO (0.99)	BO (0.99)	BO (-1.00)	BO (0.44)	BO (-0.99)	BO (0.24)	BO (-0.98)	BO (NDC)	BO (0.15)
		BG (0.89)	BG (0.12)	BG (0.80)	BG (0.39)	BG (0.70)	BG (0.33)	BG (-0.30)	BG (NDC)	BG (NDC)
C18:3	Negative	BO (0.99)	BO (NDC)	BO (NDC)	BO (0.66)	BO (NDC)	BO (NDC)	BO (-1.00)	BO (NDC)	BO (NDC)
		BG (-0.52)	BG (NDC)	BG (NDC)	BG (-0.82)	BG (NDC)	BG (NDC)	BG (-0.26)	BG (NDC)	BG (NDC)
C18:2	Negative	BO (0.90)	BO (0.71)	BO (0.50)	BO (0.87)	BO (-0.90)	BO (0.69)	BO (-0.93)	BO (NDC)	BO (0.76)
		BG (-0.78)	BG (-0.53)	BG (0.93)	BG (0.67)	BG (-0.90)	BG (-0.65)	BG (0.99)	BG (NDC)	BG (NDC)
C18:1	Negative	BO (0.98)	BO (0.30)	BO (-0.70)	BO (0.71)	BO (0.02)	BO (-0.47)	BO (-0.99)	BO (NDC)	BO (-0.55)
		BG (-0.99)	BG (0.39)	BG (0.29)	BG (0.22)	BG (0.90)	BG (0.82)	BG (0.80)	BG (NDC)	BG (NDC)
C18:0	Negative	BO (-0.98)	BO (0.33)	BO (-0.30)	BO (-0.73)	BO (-0.62)	BO (-0.87)	BO (0.99)	BO (NDC)	BO (-0.91)
		BG (NDC)	BG (-0.77)	BG (0.90)	BG (NDC)	BG (-1.00)	BG (-0.69)	BG (NDC)	BG (NDC)	BG (NDC)
C19:1	NDT	BO (NDC)	BO (NDC)	BO (-0.80)	BO (NDC)	BO (NDC)	BO (0.77)	BO (NDC)	BO (NDC)	BO (0.71)
		BG (NDC)	BG (NDC)	BG (NDC)	BG (NDC)	BG (NDC)	BG (NDC)	BG (NDC)	BG (NDC)	BG (NDC)
C19:0	NDT	BO (0.99)	BO (NDC)	BO (NDC)	BO (0.69)	BO (NDC)	BO (NDC)	BO (-1.00)	BO (NDC)	BO (NDC)
		BG (NDC)	BG (NDC)	BG (0.27)	BG (NDC)	BG (NDC)	BG (0.83)	BG (NDC)	BG (NDC)	BG (NDC)
C20:4	NDT	BO (NDC)	BO (-0.26)	BO (0.40)	BO (NDC)	BO (0.55)	BO (-0.98)	BO (NDC)	BO (NDC)	BO (-0.96)
		BG (NDC)	BG (0.86)	BG (0.08)	BG (NDC)	BG (1.00)	BG (0.92)	BG (NDC)	BG (NDC)	BG (NDC)
C20:3	NDT	BO (-0.35)	BO (-0.88)	BO (1.00)	BO (0.58)	BO (0.99)	BO (-0.24)	BO (0.28)	BO (NDC)	BO (-0.14)
		BG (NDC)	BG (0.25)	BG (0.86)	BG (NDC)	BG (-0.40)	BG (-0.75)	BG (NDC)	BG (NDC)	BG (NDC)
C20:2	NDT	BO (NDC)	BO (NDC)	BO (NDC)	BO (NDC)	BO (NDC)	BO (NDC)	BO (NDC)	BO (NDC)	BO (NDC)
		BG (NDC)	BG (-0.67)	BG (0.83)	BG (NDC)	BG (-1.00)	BG (-0.79)	BG (NDC)	BG (NDC)	BG (NDC)
C20:1	Negative	BO (NDC)	BO (NDC)	BO (NDC)	BO (NDC)	BO (NDC)	BO (NDC)	BO (NDC)	BO (NDC)	BO (NDC)
		BG (NDC)	BG (1.00)	BG (1.00)	BG (NDC)	BG (0.80)	BG (-0.32)	BG (NDC)	BG (NDC)	BG (NDC)
C20:0	Negative	BO (0.99)	BO (-0.63)	BO (-0.80)	BO (0.46)	BO (0.35)	BO (0.81)	BO (-0.98)	BO (NDC)	BO (0.76)
		BG (-0.40)	BG (-0.41)	BG (-0.97)	BG (-0.89)	BG (-0.90)	BG (0.08)	BG (-0.38)	BG (NDC)	BG (NDC)
C22:0	NDT	BO (0.89)	BO (-0.95)	BO (0.10)	BO (0.87)	BO (1.00)	BO (0.95)	BO (-0.92)	BO (NDC)	BO (0.97)
		BG (-0.58)	BG (NDC)	BG (0.94)	BG (-0.78)	BG (NDC)	BG (-0.62)	BG (-0.19)	BG (NDC)	BG (NDC)
C24:0	NDT	BO (0.66)	BO (NDC)	BO (NDC)	BO (0.99)	BO (NDC)	BO (NDC)	BO (-0.71)	BO (NDC)	BO (NDC)
		BG (0.06)	BG (NDC)	BG (-0.86)	BG (0.99)	BG (NDC)	BG (-0.21)	BG (0.68)	BG (NDC)	BG (NDC)
C26:0	NDT	BO (0.99)	BO (NDC)	BO (NDC)	BO (0.69)	BO (NDC)	BO (NDC)	BO (-0.99)	BO (NDC)	BO (NDC)
		BG (-0.84)	BG (NDC)	BG (-0.90)	BG (-0.48)	BG (NDC)	BG (-0.13)	BG (0.20)	BG (NDC)	BG (NDC)
SUM		BO (-0.91)	BO (0.34)	BO (-0.80)	BO (-0.85)	BO (-0.62)	BO (-0.35)	BO (0.94)	BO (NDC)	BO (-0.44)
		BG (-0.87)	BG (0.64)	BG (0.68)	BG (-0.43)	BG (0.00)	BG (0.49)	BG (0.25)	BG (NDC)	BG (NDC)

Correlation coefficients (r) are presented in brackets. BO–*B*. *orientalis*; BG–*B*. *germanica*, NDC–not detected; NDT–not determined

* data concerning compounds’ effects on the *in vitro* growth, sporulation and virulence of *C*. *coronatus* are from [15].

**Table 6:** Correlation between the concentration of internal FFAs of two cockroach species and the efficiency of *C*. *coronatus* proteases, chitinases and lipases in degrading the cockroach cuticle.

FFA	Effect on *C*. coronatus^*^	Proteases			Chitinases			Lipase		
		oothecae	wings	thoraces	oothecae	wings	thoraces	oothecae	wings	thoraces
C6:0	Negative	BO (0.66)	BO (0.67)	BO (0.06)	BO (-0.25)	BO (-0.40)	BO (0.96)	BO (-0.60)	BO (NDC)	BO (0.98)
		BG (-0.88)	BG (0.96)	BG (-0.80)	BG (-0.41)	BG (0.57)	BG (0.85)	BG (0.27)	BG (NDC)	BG (NDC)
C7:0	Negative	BO (0.89)	BO (NDC)	BO (NDC)	BO (0.12)	BO (NDC)	BO (NDC)	BO (-0.90)	BO (NDC)	BO (NDC)
		BG (-0.79)	BG (NDC)	BG (-0.30)	BG (-0.56)	BG (NDC)	BG (1.00)	BG (0.11)	BG (NDC)	BG (NDC)
C8:0	Negative	BO (0.91)	BO (-0.76)	BO (-0.40)	BO (0.16)	BO (0.93)	BO (-0.80)	BO (-0.90)	BO (NDC)	BO (-0.85)
		BG (-0.90)	BG (-0.20)	BG (1.00)	BG (-0.38)	BG (-0.76)	BG (-0.32)	BG (0.30)	BG (NDC)	BG (NDC)
C9:0	Negative	BO (0.94)	BO (-0.75)	BO (1.00)	BO (0.26)	BO (0.92)	BO (-0.25)	BO (-0.90)	BO (NDC)	BO (-0.16)
		BG (-0.86)	BG (-0.43)	BG (-1.00)	BG (-0.45)	BG (-0.89)	BG (0.39)	BG (0.23)	BG (NDC)	BG (NDC)
C10:0	Negative	BO (1.00)	BO (NDC)	BO (-0.97)	BO (0.59)	BO (NDC)	BO (0.44)	BO (1.00)	BO (NDC)	BO (0.36) BG (NDC)
		BG (-0.99)	BG (NDC)	BG (NDC)	BG (-0.10)	BG (NDC)	BG (NDC)	BG (0.56)	BG (NDC)	
C12:0	Negative	BO (0.38)	BO (0.58)	BO (0.95)	BO (-0.55)	BO (-0.28)	BO (0.08)	BO (-0.30)	BO (NDC)	BO (0.17)
		BG (-0.81)	BG (0.28)	BG (-1.00)	BG (-0.53)	BG (-0.37)	BG (0.44)	BG (0.14)	BG (NDC)	BG (NDC)
C14:0	Negative	BO (0.86)	BO (0.66)	BO (0.19)	BO (0.05)	BO (-0.87)	BO (-1.00)	BO (-0.80)	BO (NDC)	BO (-1.00)
		BG (-0.87)	BG (0.88)	BG (-1.00)	BG (-0.44)	BG (0.39)	BG (0.01)	BG (0.24)	BG (NDC)	BG (NDC)
C15:0	Positive	BO (0.76)	BO (0.54)	BO (-0.67)	BO (-0.11)	BO (-0.78)	BO (-0.57)	BO (-0.70)	BO (NDC)	BO (-0.65)
		BG (-0.26)	BG (-0.98)	BG (-0.90)	BG (-0.95)	BG (-0.66)	BG (-0.16)	BG (-0.52)	BG (NDC)	BG (NDC)
C16:1	Negative	BO (0.79)	BO (-1.00)	BO (-0.98)	BO (-0.07)	BO (0.92)	BO (0.44)	BO (-0.70)	BO (NDC)	BO (0.35)
		BG (-0.84)	BG (-0.04)	BG (-0.30)	BG (-0.49)	BG (-0.65)	BG (1.00)	BG (0.18)	BG (NDC)	BG (NDC)
C16:0	Negative	BO (0.75)	BO (-0.75)	BO (-0.92)	BO (-0.13)	BO (0.50)	BO (-0.16)	BO (-0.70)	BO (NDC)	BO (-0.25)
		BG (-0.80)	BG (0.42)	BG (-1.00)	BG (-0.55)	BG (-0.23)	BG (0.04)	BG (0.11)	BG (NDC)	BG (NDC)
C17:1	Negative	BO (0.72)	BO (-0.62)	BO (-0.08)	BO (-0.17)	BO (0.84)	BO (-0.95)	BO (-0.70) BG (NDC)	BO (NDC)	BO (-0.98)
		BG (NDC)	BG (-0.89)	BG (0.00)	BG (NDC)	BG (-0.42)	BG (-0.95)		BG (NDC)	BG (NDC)
C17:0	Negative	BO (0.84)	BO (0.06)	BO (-0.63)	BO (0.03)	BO (-0.38)	BO (0.90)	BO (-0.80)	BO (NDC)	BO (0.86)
		BG (-0.64)	BG (-0.62)	BG (-1.00)	BG (-0.73)	BG (0.00)	BG (0.13)	BG (-0.11)	BG (NDC)	BG (NDC)
C18:3	Negative	BO (0.31)	BO (NDC)	BO (NDC)	BO (-0.62)	BO (NDC)	BO (NDC)	BO (-0.20)	BO (NDC)	BO (NDC)
		BG (-0.82)	BG (NDC)	BG (NDC)	BG (-0.52)	BG (NDC)	BG (NDC)	BG (0.15)	BG (NDC)	BG (NDC)
C18:2	Negative	BO (0.61)	BO (0.00)	BO (-0.48)	BO (-0.31)	BO (0.32)	BO (-0.74)	BO (-0.60)	BO (NDC)	BO (-0.80)
		BG (-0.84)	BG (-0.81)	BG (0.90)	BG (-0.49)	BG (-0.27)	BG (-0.76)	BG (0.19)	BG (NDC)	BG (NDC)
C18:1	Negative	BO (0.72)	BO (1.00)	BO (0.21)	BO (-0.16)	BO (-0.96)	BO (0.90)	BO (-0.70)	BO (NDC)	BO (0.94)
		BG (-0.78)	BG (-0.90)	BG (-1.00)	BG (-0.58)	BG (-0.44)	BG (0.31)	BG (0.08)	BG (NDC)	BG (NDC)
C18:0	Negative	BO (0.53) BG (NDC)	BO (-0.71)	BO (0.88)	BO (-0.40) BG (NDC)	BO (0.44)	BO (-0.26)	BO (-0.50) BG (NDC)	BO (NDC)	BO (-0.35)
			BG (0.91)	BG (-1.00)		BG (0.46)	BG (0.23)		BG (NDC)	BG (NDC)
C19:1	NDT	BO (NDC)	BO (NDC)	BO (0.07) BG (NDC)	BO (NDC)	BO (NDC)	BO (0.96) BG (NDC)	BO (NDC)	BO (NDC)	BO (0.98)
		BG (NDC)	BG (NDC)		BG (NDC)	BG (NDC)		BG (NDC)	BG (NDC)	BG (NDC)
C19:0	NDT	BO (0.92) BG (NDC)	BO (NDC)	BO (0.93) BG (NDC)	BO (0.20) BG (NDC)	BO (NDC)	BO (0.15) BG (NDC)	BO (-0.90) BG (NDC)	BO (NDC)	BO (0.24 BG (NDC)
			BG (NDC)			BG (NDC)			BG (NDC)	
C20:4	NDT	BO (0.47) BG (NDC)	BO (NDC)	BO (0.69)	BO (-0.46) BG (NDC)	BO (NDC)	BO (-0.86)	BO (-0.40) BG (NDC)	BO (NDC)	BO (-0.81)
			BG (0.53)	BG (-0.40)		BG (0.94)	BG (-0.76)		BG (NDC)	BG (NDC)
C20:3	NDT	BO (0.63)	BO (-0.30)	BO (-0.76)	BO (-0.29)	BO (-0.02)	BO (0.81)	BO (-0.60)	BO (NDC)	BO (0.75)
		BG (0.19)	BG (-0.89)	BG (-0.60)	BG (-0.99)	BG (-0.98)	BG (0.93)	BG (-0.84)	BG (NDC)	BG (NDC)
C20:2	NDT	BO (0.85)	BO (NDC)	BO (-0.89) BG (NDC)	BO (0.03) BG (NDC)	BO (NDC)	BO (0.64) BG (NDC)	BO (-0.80) BG (NDC)	BO (NDC)	BO (0.57)
		BG (NDC)	BG (NDC)			BG (NDC)			BG (NDC)	BG (NDC)
C20:0	Negative	BO (0.98)	BO (0.22)	BO (-0.86)	BO (0.38)	BO (0.11) BG (NDC)	BO (0.70)	BO (-1.00)	BO (NDC)	BO (0.63)
		BG (-0.29)	BG (NDC)	BG (-0.30)	BG (-0.93)		BG (1.00)	BG (-0.48)	BG (NDC)	BG (NDC)
C22:0	NDT	BO (0.49)	BO (NDC)	BO (1.00)	BO (-0.44)	BO (NDC)	BO (-0.13)	BO (-0.40)	BO (NDC)	BO (-0.04)
		BG (-0.42)	BG (NDC)	BG (-1.00)	BG (-0.88)	BG (NDC)	BG (0.05)	BG (-0.36)	BG (NDC)	BG (NDC)
C24:0	NDT	BO (0.76)	BO (NDC)	BO (NDC)	BO (-0.11)	BO (NDC)	BG (0.52) BG (NDC)	BO (-0.70)	BO (NDC)	BO (NDC)
		BG (-0.99)	BG (NDC)	BG (-1.00)	BG (-0.09)	BG (NDC)		BG (0.57)	BG (NDC)	BG (NDC)
C26:0	NDT	BO (0.95)	BO (NDC)	BO (NDC)	BO (0.27)	BO (NDC)	BO (NDC)	BO (-0.90)	BO (NDC)	BO (NDC)
		BG (NDC)	BG (NDC)	BG (NDC)	BG (NDC)	BG (NDC)	BG (NDC)	BG (NDC)	BG (NDC)	BG (NDC)
SUM		BO (0.68)	BO (0.93)	BO (-0.38)	BO (-0.22)	BO (-0.77)	BO (0.99)	BO (-0.60)	BO (NDC)	BO (0.97)
		BG (-0.80)	BG (-0.91)	BG (-1.00)	BG (-0.55)	BG (-0.47)	BG (0.13)	BG (0.12)	BG (NDC)	BG (NDC)

Correlation coefficients (r) are presented in brackets. BO–*B*. *orientalis*; BG–*B*. *germanica*, NDC–not detected, NDT–not determined

*data concerning compounds’ effects on the in vitro growth, sporulation and virulence of *C*. *coronatus* are from [15]

Chitin degradation negatively correlated to a lesser extent with C18:3 in oothecae (*B*. *orientalis* r = -0.62, *B*. *germanica* r = -0.52) (Table 6). In both cockroach species, C18:3 was detected only in the oothecae. No positive or negative correlations were found between cuticular FFA content and efficiency of fungal lipases. Internal C20:3 was negatively correlated with lipid degradation in oothecae. A slightly less distinct negative correlation was also observed for C15:0 (*B*. *orientalis* r = -0.70, *B*. *germanica* r = -0.52) (Tables 5 and 6).

## Discussion

Although chemical pesticides are among the most popular methods of controlling cockroach infestations, their disadvantages have spurred the search for new strategies, including the use of entomopathogenic fungi [34,37,38]. *C*. *coronatus* is a cosmopolitan soil fungus that selectively attacks various insect species [39]. Our findings indicate that *B*. *orientalis* and *B*. *germanica* are not susceptible to infection by *C*. *coronatus*, but not to infection by other entomopathogenic fungi, such as *Metarhizium anisopliae*, *Beauveria bassiana* and *Purpureocillium lilacinum* [35,40–42]

The mycelia of *C*. *coronatus* cultivated *in vitro* secrete a plethora of enzymes, however, the activities of fungal enzymes measured *in vitro* are not necessarily correlated with their importance in the infection process occurring in nature [15,43–46]. The enzymatic cocktail released by *C*. *coronatus* mycelia degrades cuticle samples from susceptible insects far more effectively than those from resistant species and/or developmental stages [11,28,47]. Similar differences were observed in the present study for *B*. *orientalis* and *B*. *germanica*, particularly regarding the digestion of oothecae proteins by fungal proteases; this might indicate higher levels of total protein in *B*. *germanica* oothecae than in *B*. *orientalis*, or of proteins susceptible to digestion by *C*. *coronatus* proteases.

^13^C –NMR spectroscopy revealed higher levels of proteins in the cuticle of *B*. *germanica* oothecae than for *B*. *orientalis* [48]; this may be due to the higher protein requirement of developing nymphs [49] and/or differences in their physiology: *B*. *orientalis* females deposit oothecae as soon as they are formed while *B*. *germanica* females retain the oothecae until nymphs are ready to hatch.

In contrast to the oothecae, the two cockroach species released similar, low amounts of amino acids, suggesting their protein content was low in wings and had similar protein compositions. *C*. *coronatus* proteases have been found to be highly effective against the wings proteins of four fly species (*L*. *sericata*, *C*. *vicina*, *C*. *vomitoria*, *M*. *domestica*) and those of *G*. *mellonella* [28,47]. It could suggest a lower content of degradable proteins in the cockroach wings compared to other insects we have studied in terms of efficiency of cuticular protein digestion by *C*. *coronatus* proteases. Similar high concentrations of amino acids were released from the digested thoraces of *B*. *orientalis* and *B*. *germanica*, suggesting a high abundance of similar proteins. Taken together, our findings suggest that the protein composition of the cuticle varies considerably across the body of the insect.

In contrast, no significant differences were found in the effectiveness of chitin hydrolysis in all samples of both cockroach species, indicating no species-specific variation and similar spatial distribution of chitin in the bodies. However, the *C*. *coronatus* chitinolytic enzymes demonstrated greater efficiency against both cockroach species compared to four fly species [28] and the wax moth [47] suggesting higher levels of chitin in cockroach cuticles. *N*-glucosamine was released from oothecae incubated with *C*. *coronatus* enzymatic cocktail containing chitinases, thus confirming the presence of chitin. It has long been assumed that chitin was absent from oothecae [50], however, this belief has been challenged by recent studies [48,51–53].

The lipases present in the *C*. *coronatus* enzyme cocktail demonstrated less hydrolytic activity against the cuticle samples than the proteases and chitinases; FFAs were only released from the oothecae of both species and thoraces of *B*. *orientalis*. Similar results have been noted against the previously described four fly species and *G*. *mellonella* [28,47]. It appears that in *C*. *coronatus*, lipases play a lesser role in the development of an infection to that of proteases and chitinases, in contrast with the pivotal role of lipolytic activity during *M*. *anisopliae* infection [27].

GC-MS identified several FFAs in *B*. *orientalis* and *B*. *germanica* cuticle samples, these being odd-numbered FFAs: seven saturated (C5:0, C7:0, C9:0, C13:0, C15:0, C17:0, C19:0) and three unsaturated (C15:1, C17:1, C19:1). The presence of odd-numbered FFAs on the surface of insects is rare. Traces of pentadecenoic acid (C15:1) were identified in the cuticular lipids of *Acyrthosiphon pisum* [54] and chlorpyrifos-treated *B*. *germanica* males [55]. This FFA is also characteristic for the cuticle of *C*. *vomitoria* males [36] and *Nezara viridula* infected by the plant pathogenic fungus *Paecilomyces spp* [56]. In present studies C15:1 was detected only in cuticular extracts from *B*. *germanica* oothecae.

The cuticular and internal FFAs identified in this work are similar to those previously identified for *B*. *orientalis* and *B*. *germanica* [38,55,57]. Slight discrepancies in the presence and quantity of individual FFAs result from variation in the use of GC-MS instruments, extraction and derivatization procedures, and from the different starting materials: we used both males and females pooled together, while Paszkiewicz et al. examined *B*. *orientalis* females and *B*. *germanica* males only. Most previously examined insect species indicate higher abundance of FFAs in the internal lipids than in cuticular lipids [16,18,36]; however, *Chorthippus brunneus* appears to be an exception, as are the present results [58]. Higher amounts of FFAs were found in wings and thoraces (g^-1^ of tissue) than in the whole body of adults (g^-1^ insect body); this could be due to the high number of these light body parts (*B*. *orientalis*: 539 wings and 274 thoraces; *B*. *germanica* 377 wings and 186 thoraces) required to extract sufficient amounts of lipids for GC-MS analyses. *B*. *orientalis* display a clear wing dimorphism; the present study used equal amounts of reduced and leathery female wings and longer, membranous male wings.

Species specific differences in cuticular FFA profiles were found between cockroach species: C4:0 and C5:0 was present only *in B*. *germanica*, while C19:1 was found only in the thoraces of *B*. *orientalis* indicating an uneven spatial distribution. The physiological functions of these FFAs in cockroaches remain unknown. While C18:3 was found solely in the oothecae of both species, its exact role is unknown; however, it is likely to protect against fungal attack as C18:3 inhibits *C*. *coronatus* growth and the growth and germination of *B*. *bassiana* and *Paecilomyces fumosoroseus* [21,59]. The origin of C13:0, detected only in *B*. *orientalis* oothecae remains obscure. The same applies to C14:1 and C15:1, found only in *B*. *germanica* oothecae. C13:0, C14:1 and C15:1 have demonstrated antifungal activity against *C*. *coronatus* and several pathogenic fungi [15,60]. Eleven FFAs (C6:0, C7:0, C8:0, C9:0, C10:0, C12:0, C16:0, C18:1, C18:2, C18:3 and C20:0) known to inhibit key factors determining the ability of *C*. *coronatus* to infect insects, i.e. hyphal growth, sporulation and virulence [15], were found in the oothecae of both species, indicating multiple investments in protecting cockroach eggs and developing offspring. The cockroach ootheca is formed from the secretions of two colleterial glands containing proteins, enzymes and catechol derivatives [53]. The method of delivery of lipids to the ootheca is poorly understood [61], and the presence and amount of each cuticular FFA is the result of a number of poorly-understood processes of synthesis, degradation and distribution in the insect body and transportation to the target sites [28]

The efficiency in degrading cockroach cuticle samples by *C*. *coronatus* proteases was found to be negatively correlated with concentrations of C6:0, C9:0, C10:0, C16:0 and C20:0. This suggests that these FFAs may play a protective role against fungal assault. However, this inference is weakened by the positive correlations found between fungal protease efficiency and concentrations of C6:0 in the wings, C12:0 and C20:3 in the thoraces, and C17:0 in the oothecae.

In the case of *C*. *coronatus*, the role of chitinases is even more complex, as both negative and positive correlations were found regarding the same FFAs, but these differed according to body part. Obviously, more experiments are necessary to demonstrate the impact of each FFA detected in cockroach cuticle on the activity of fungal enzymes engaged in the initial stage of fungal attack.

The present study partly elucidates the mechanisms underlying the non-susceptibility of two species of cockroaches, *B*. *germanica* and *B*. *orientalis* to fungal infection and highlights the role of FFAs in that process. Further studies on the role played by cuticular lipids in the interaction between the invading fungus and the insect host will shed greater light on the complexity of the infection process.

## Materials and methods

### Insects

*B*. *orientalis* and *B*. *germanica* were cultured in the laboratory at 25°C, 70% relative humidity (RH), and a 12:12-hour photoperiod. The insects were cultured on standard rodent food (Agropol, Poland). For cuticle preparations, both adults and oothecae were used.

### Entomopathogenic fungus

The entomopathogenic fungus was *C*. *coronatus* (isolate no. 3491), originally isolated from *Dendrolaelaps* spp. (Mesostigmata: Digamasellidae), obtained from the collection of Professor Bałazy (Polish Academy of Sciences, Research Centre for Agricultural and Forest Environment, Poznań, Poland). The fungus was maintained in 90 mm Petri dishes at 20°C in a 12:12-hour light/dark cycle to stimulate sporulation [62] on Sabouraud agar medium (SAM). The medium was supplemented by homogenized *G*. *mellonella* larvae to a final concentration of 10% wet weight. This addition enhances sporulation and virulence of the SAM cultures of *C*. *coronatus*. At seven days, conidia were harvested by flooding the plates with sterile water; 100μL portions of suspension, each containing approximately 50 conidia, were taken for inoculations.

To obtain the mixture of fungal enzymes to hydrolyze the insect cuticle, *C*. *coronatus* was cultivated at 20°C in 500-ml Erlenmeyer flasks containing 250ml of minimal medium as described by Bania and co-workers but without shaking [43]. After three weeks, the mycelia were removed by filtration through Whatman no. 1 filter paper. The cell-free filtrates were assayed for their protein concentrations and protease, chitinase and lipase activities, and taken for *in vitro* hydrolysis of cockroach cuticle preparations.

The same *C*. *coronatus* cell-free filtrate was used in studies of cuticle hydrolysis in four medically-important fly species and *Galleria mellonella* [28,47].

### Infection of insects with *C*. *coronatus*

*B*. *orientalis* and *B*. *germanica* adults were exposed for 24 hours at 20˚C to fully-grown and sporulating *C*. *coronatus* colonies, around 10 per Petri dish. Controls were exposed for 24 hours to sterile Sabouraud agar medium. After exposure, the insects were transferred to new, clean Petri dishes with appropriate food, and observed for seven days.

Oothecae were exposed in the same way within 24 hours of being laid by the females. The effectiveness of fungus penetration into the oothecae and their impact on developing insects was measured as the percentage of larvae that were dead within three days of hatching.

### Cuticle preparation

Frozen adults of *B*. *orientalis* and *B*. *germanica* were briefly (5–10 min) rinsed in tap water and then thoroughly dried with a paper towel. The wings were dissected, and the remnants of the muscles were removed. The cuticles were dissected from thoraces in 10 mM ice-cold Tris-HCl buffer (pH 7.0) and carefully cleaned of remnants of fat body, muscles and other tissues. Empty oothecae were cleaned inside to remove the remnants left by eggs and hatching larvae. All prepared cuticle pieces were washed three times in 10mM ice-cold Tris-HCl buffer (pH 7.0), allowed to dry on ice-cold towels and stored at −20°C until use.

### Enzymatic assays

Elastase, *N*-acetylglucosaminidase (NAGase), chitinase and lipase activity were measured in *C*. *coronatus* cell-free filtrates according to Boguś and co-workers [28]. Measurements were taken spectrophotometrically and spectrofluorimetrically (BioTek Synergy HT, USA) in 96-well polystyrene plates using suitable synthetic substrates (Merck, Germany). Elastolytic activity was measured using *N-*succinyl-alanine-alanine-proline-leucine-p-nitroanilide in 100mM Tris-HCl buffer containing 20mM CaCl_2_ (pH 8.0). The reactions were performed in plate wells containing 2 μl of cell-free filtrate comprising fungal enzymes, 0.5mM final substrate concentration, and reaction buffer to a final volume of 200 μl. The reaction was started by the addition of the substrate, and readings were taken at A_410_ to create a progress curve. Chitobiosidase activity was measured using a 0.003mM final concentration of 4-methylumbelliferyl 𝛽-D-N-N′-diacetylchitobioside in 50mM Tris-HCl buffer (pH 7.0). Fluorescence was read at Ex = 340 nm and Em = 450 nm. NAGase activity was measured using a 0.3mM final concentration of 4-nitrophenyl-N-acetyl-𝛽-D-glucosaminide in 10mM Tris-HCl buffer (pH 7.0). Absorbance was read at 405 nm. Lipase activity was measured using a 0.01mM final concentration of 4-methylumbelliferyl oleate in 50mM Tris-HCl buffer (pH 10.0). Fluorescence was read at Ex = 360 nm and Em = 450 nm.

### Determination of protein concentration

The protein concentration of the cell-free filtrate of *C*. *coronatus* was determined with the Bio-Rad Protein Assay (USA), according to Bradford. Briefly, an acidic dye (Coomassie Brilliant Blue) was added to the protein solution, and the absorbance was measured at 595 nm with a microplate reader. Absorbances were measured using BioTek Synergy HT. Bovine serum albumin (BSA) was used as the standard.

### Hydrolysis of insect cuticle incubated with cell-free filtrate of *C*. *coronatus*

The insect cuticle samples were divided into 50 mg portions, ground in liquid nitrogen and then washed four times in 10 mM Tris-HCl buffer (pH 7.0); 10 mg of ground cuticle was suspended in 1ml of the 10mM Tris-HCl buffer (pH 7.0), 800 μl of which was mixed with 228 μl of the *C*. *coronatus* cell-free filtrate containing elastase, NAGase, chitobiosidase and lipase. The reaction mixture was incubated for eight hours at 30°C. The reaction cocktail was divided into 20 μl portions and immediately frozen to stop further hydrolysis. Two negative controls were added, one consisting of reaction buffer with 1 mg of cuticle but without the cell-free *C*. *coronatus* filtrate (C1), and the other consisting of buffer with *C*. *coronatus* filtrate but without the insect cuticle (C2). The free amino acids produced by hydrolysis of the cuticle by proteases were measured according to Adler-Nissen, with some modifications [63]. The samples and the controls were mixed with 0.1% picrylsulfonic acid (Merck, Germany) and read at A_340_. The absorbance of the negative controls was subtracted from the samples. The amounts of *N-*glucosamine released by chitinase hydrolysis were measured using the D-glucosamine Assay Kit (Megazyme, Ireland) according to the producer’s manual. The concentrations of free fatty acids (FFAs) released by lipases were determined with the use of the EnzymChrom TM Free Fatty Acid Assay Kit (BioAssay Systems, USA). Three independent replications of all procedures were performed. The hydrolytic efficiency of the fungal enzymes was calculated per mg of cuticle. No determination of cuticle protein, chitin and lipid content was not performed due to due to the amounts of insect-derived material being insufficient.

### Extraction of free fatty acids (FFAs)

Cuticular and internal lipid components of insects were extracted, separated and analysed by GC-MS. Whole adults, oothecae, wings and thoraces isolated from adults (mass in Table 2) were extracted first in 20 ml of petroleum ether (Merck, Germany) for 5 min (extract I) and then again in 20 ml of dichloromethane (Merck, Germany) for 5 min (extract II) to yield cuticular lipids. The insects and cuticle preparations were sonicated with dichloromethane to produce Extract III containing internal lipids. The extracts were placed in glass flasks and evaporated under nitrogen.

### Derivatization method

Trimethylsilyl esters (TMS) of FFAs were obtained by adding 100 μl of a BSTFA: TMCS mixture (99:1) (Merck, Germany) to 1 mg of sample and heating for 1h at 100°C. The TMS of fatty acids were then analysed by GC-MS.

### GC-MS analyses

The analyses were carried out on a GCMS-QP2010 with mass detector (Shimadzu, Japan). Helium was used as the carrier gas at a column head pressure of 65.2 kPa. A DB-5 MS (Zebron, Phenomenex, USA) column was used (thickness 0.25 μm, length 30 m, diameter 0.25 μm). The column oven temperature cycle was 80^º^C for 3 min then 80°C to 310ºC at 4°C/min; the final temperature was then held for 10 min. The ion source temperature was 200°C and the interface temperature was 310^º^C. Split mode was used with a split ratio of 10. All compounds were identified based on fragmentation patterns and mass-to-charge ions of the TMS derivatives and the NIST 11 library. The mass spectrum of the fatty acid trimethylsilyl esters comprised M+ (molecular ion), [M-15]+, and fragment ions at m/z 117, 129, 132, and 145. GC analysis used the 19-methylarachidic acid (1 mg/ml; Merck, Germany) as an internal standard (IS). The contents were calculated from the relative peak areas that were compared to the IS peak area and expressed as a percentage (%, w/w) of total extracts. Response factors of one were assumed for all constituents.

### Statistics

The findings were tested by the parametric t-test and one-way analysis of variance (ANOVA), where appropriate. Tukey’s test was used for *post hoc* analysis. Each test was performed separately. All analyses were performed using Statistica 6 software (StatSoft Polska, Poland). Differences were significant at p<0.05.

## Supporting information

S1 Table The resistance of *B. orientalis* and *B. germanica* to fungal infection–raw data. (XLSX)

S2 Table Hydrolysis of cuticular protein, chitin and lipid by *C. coronatus* enzymes–raw data. (XLSX)

S3 Table GC-MS analysis of cuticular lipids by *C. coronatus* enzymes–raw data. (XLSX)
